# A New Title and a New Look

**Published:** 1999

**Authors:** Enoch Gordis

**Affiliations:** Director National Institute on Alcohol Abuse and Alcoholism

This issue of *Alcohol Health & Research World* introduces both a new title and a new look. These changes reflect a series of improvements that have occurred in the journal’s format and content over the past several years that are designed to maintain and improve the journal’s relevance and utility to our readers. Since its inception in the 1970s, the journal has evolved from a general interest magazine to an internationally known peer-review science journal. This evolution reflects the changing mission of the National Institute on Alcohol Abuse and Alcoholism (NIAAA) from a general purpose alcohol agency to an internationally respected research Institute. It also reflects the changing nature of the alcohol field and the growing need for science-based information to better understand and improve our ability to prevent, treat, and reduce the problems associated with alcohol abuse and alcoholism.

In recent years, we have taken great care to assure that the material presented in the journal is informative as well as scientifically sound. This challenge has included presenting technical findings in ways that are interesting and useful to the wide range of disciplines and agendas that comprise our audience, including educators, counselors, clinicians, researchers, and policymakers. As we have progressed to a fully refereed journal, we also have become aware that the title of the journal does not truly reflect the full scope and substance of the information presented in each issue. With this in mind, we recently invited readers and members of the alcohol research field to submit ideas for a new title commensurate with the evolving “ flavor” of the journal. With this issue, we are pleased to announce that a new title has been selected. Please be welcomed to *Alcohol Research & Health.*

Though not very different from our original name, we feel that this new title will give readers a more accurate sense of our journal’s mission. We are about research—that is, the information we feature is evidence based and fully reviewed by professionals in the field. We also are about health in that our ultimate goal is to disseminate the science that informs the practice of caring for and about people affected by alcohol problems.

In launching the new title, we also have instituted some minor design changes. These modifications are intended to make the material presented in the journal more “reader friendly.” We hope you like the new title and the corresponding new design. We look forward to continuing to provide the most up-to-date research findings on topics that are important in alcohol research.

